# Differential relationships between discount rates and health behaviors in an ethnically diverse college sample

**DOI:** 10.3389/fpubh.2022.943499

**Published:** 2022-08-09

**Authors:** Natashia Bibriescas, Katherine Wainwright, Rebecca Thomas, Victoria Lopez, Paul Romanowich

**Affiliations:** ^1^Department of Educational Psychology, The University of Texas at Austin, Austin, TX, United States; ^2^Department of Psychology, The University of Texas at San Antonio, San Antonio, TX, United States; ^3^Ecampus Research Unit, Oregon State University, Corvallis, OR, United States; ^4^Department of Psychology, Gonzaga University, Spokane, WA, United States

**Keywords:** delay discounting, social discounting, alcohol consumption, STI, exercise

## Abstract

Previous research has demonstrated associations between delay discount rate and engagement in several health behaviors. The delay discount rate is also inversely associated with social discount rates, a putative measure for sharing. However, there is little research that examines whether delay and social discount rates are differentially associated with health behavior engagement, and even less research examining the impact of ethnicity on these relationships. This study investigated whether delay and/or social discount rates predict three health behaviors varying in sociality: sexually transmitted infection (STI) testing, alcohol consumption and exercise frequency in an ethnically diverse university sample. The results showed that neither delay nor social discount rate significantly predicted alcohol consumption and exercise frequency. However, increasing social discount rates (i.e., decreased sharing) was associated with a decreased likelihood to be tested for STIs. Ethnicity significantly contributed to two models, indicating differences in STI testing and alcohol consumption across ethnicities. Ethnic differences in these health behaviors were consistent with many previous health behavior studies, suggesting a profitable way to research cultural contingencies and test the reliability of the ethnically diverse data. These findings indicate that the social discount rate is differentially associated with health behaviors with more social aspects (i.e., health behaviors related to sex) in college students.

## Introduction

Delay discounting assesses an individual's preference between smaller immediate rewards and larger delayed rewards, such as money (1, 2). Reward value decreases as delay increases (3). Historically, impulsivity has been defined as a person's ability to regulate and control impulses and urges (4). Therefore, delay discounting was used as a purported measure of impulsivity where more impulsive individuals preferred the smaller more immediate rewards rather than waiting for a larger delayed reward (5). However, the term “impulsivity” has not fared well as a psychological construct (6). That said, there is evidence that excessive delay discounting is a trans-disease process, cutting across many different problematic health behaviors (7). That is, the ability to delay immediate gratification is significantly negatively related to maladaptive behavioral health choices such as increased alcohol consumption, risky sexual behaviors, smoking, gambling, and decreased exercise [for reviews see (2, 4)]. Individuals typically encounter problems related to these health behaviors when they choose to engage in those behaviors, rather than competing beneficial health behaviors (e.g., alcohol and smoking abstinence, protected sex).

Social discounting is related to delay discounting and is a putative measure for one's willingness to share, or not (i.e., selfishness), within a social network. Selfishness increases as a function of increasing social distance (8–11). Thus, increasing social discount rates indicate increasing selfishness (8). Most previous studies have demonstrated a significant relationship between delay discounting and social discounting [e.g., (9, 12)]. In these cases, as delay discount rates increase, social discount (i.e., selfishness) rates also increase [but see Igaki et al. (13) for null results].

Many important health behaviors significantly associated with delay discounting also have social aspects, even though this association is seldom studied. For example, many individuals consume alcohol and exercise in social settings, and sexually transmitted infections (STIs) necessarily require a social component. In addition, researchers have theorized that social discounting is simply an extension of delay discounting outside of the self [i.e., (14)]. Those individuals that can delay gratification (share with themselves) are more likely to share with other individuals across a range of situations, including health behaviors. Thus, it is plausible that these health behaviors would also be significantly associated with social discount rates, in addition to delay discount rates in college student samples. Below, we briefly describe associations between delay discounting and three important health behaviors (alcohol use, STIs, and exercise), and how each contains a greater or lesser social aspect. Lastly, we describe how ethnic differences may also impact associations between discounting and health behaviors, justifying further research.

### Testing for sexually transmitted infections (STIs)

STIs disproportionately affect college-aged individuals. Satterwhite et al. (15) estimated that nearly 20 million new STIs occur every year and approximately half occur in individuals aged 15–24. Despite the high incidence rate, only 12% of individuals reported being tested for an STI (16). It is estimated that STIs cost the United States $16 billion annually (17). Delay discount rates have consistently been linked to risky sexual behavior (18–20). Higher monetary delay discount rates have also been linked to sexual health-specific outcomes, all of which increase the likelihood of having an STI. This includes first sexual activity age, recent relationship infidelity, past or current pregnancy, multiple sexual partners, using less protection, and having ever had gonorrhea or chlamydia (18, 21–23). However, no research to date has extended this to determine if there is a relationship between discount rates (either delay or social) and being tested for STIs, a critical step to treating and stopping transmission. Social factors may impact individuals' decisions to get tested for STIs. For example, individuals may choose to be willfully careless (i.e., selfish) about how their sexual health affects the sexual health of others. Conversely, those more likely to share, may be more inclined to get an STI test before engaging in intercourse. The important choice of whether or not to be tested for an STI is readily measurable for sexually active individuals, and thus amenable to studying the relationship with delay and social discounting.

### Alcohol use

Approximately 55% of college students reported drinking in the past month and more than a third reported binge drinking in that timeframe (24). Alcohol use has shown a consistent relationship with delay discount rates, with increased drinking associated with higher delay discount rates (4, 25, 26). Research has also identified that alcohol consumption is associated with putting others in harm's way (24). For example, driving while intoxicated puts college-aged adults at an increased risk of an automobile accident (27). Additionally, Romanowich et al. (28) recently reported a significant relationship between a risky driving behavior [texting while driving (TWD)] and social discounting. Importantly, those individuals who reported TWD (and higher social discount rates) were also more likely to consume alcohol. Thus, it is plausible that social discount rates, or selfishness, for individuals who drink more might be higher than individuals who drink less. Romanowich and Igaki (29) reported that alcohol use was not a significant predictor of social discounting. However, alcohol use was not dichotomized as normal and unhealthy drinking, only as drinking or not. Additionally, differences in social discounting based on alcohol *quantity* consumed has yet to be assessed.

### Exercise

Approximately 40–45% of college students regularly engage in fitness activities (30). However, obesity incidence among college-aged individuals in the United States increased from 12% in 1996 to 36% in 2004 (30). Decreased physical activity in the United States is associated with $117 billion annually in healthcare costs (31). Delay discount rates are associated with less weekly physical activity [(32, 33), but see (34) for null results]. Additionally, Sofis et al. (35) experimentally showed that increased physical activity induced decreasing delay discount rates. Social influences, such as family, important others, co-exercisers, and class trainers are associated with exercise adherence (36). Individuals may feel a social obligation to others regarding their own exercise habits. Conversely, selfish individuals may choose to disregard the expectations or concerns of others and not exercise.

### Ethnic differences

Certain ethnic minorities demonstrate greater risk for adverse health outcomes that are commonly associated with health behaviors such as STI testing, alcohol use, and exercise. Both Hispanic Americans and African Americans demonstrate greater risk of STIs, alcohol abuse, alcohol dependence, and obesity when compared to White Americans and other racial minorities, such as Native Americans and Asian Americans (37–39). The disparities in health outcomes across ethnicities illustrates the necessity of investigating health behaviors *via* discounting among an ethnically diverse sample.

Many discounting studies use predominantly Caucasian samples (21, 26, 32–34, 40, 41). Prior limited research suggests certain ethnicities display different discount rates. For example, Caucasian participants demonstrated lower rates of delay discounting, than African Americans, Hispanic Americans and Native Americans (42, 43). In terms of health, one preliminary study, using eight participants, found no significant difference in delay discounting with cigarette use among Caucasian and Native Americans (42). However, as mentioned above, these examples are considerably limited by small sample sizes and should be interpreted with that in mind. Social discounting studies have primarily focused on between-culture differences [see (44)], rather than ethnic differences within one country. However, in the US there are well-documented health differences between ethnicities (39), suggesting that differential associations between health behaviors and discounting could plausibly exist.

### Hypotheses

The present study aimed to replicate previous relationships between delay discount rates and health behaviors and extend the current literature by examining social discount rates and those same health behaviors outlined above. First, based on the literature described above, we hypothesized that higher delay discount rates would predict decreased STI testing (41), increased drinks consumed per week (4), and decreased exercise per week (35). Second, given the relationship found between delay and social discounting in past research (9, 12), and social aspects of STIs, alcohol consumption (45), and exercise (36) we predicted that individuals showing higher social discount rates (i.e., increased selfishness) would have decreased STI testing, increased weekly alcohol consumption, and decreased exercise per week. Third, due to evidence of between-culture differences in discounting behaviors across countries and health disparities in the U.S. it was expected that ethnicity will differentially predict the investigated health behaviors.

## Methodology

### Participants

Participants included 395 students from the University of Texas at San Antonio (UTSA) that were recruited through their Introduction to Psychology course for a large study on important, but less studied health behaviors and their potential association with discounting rates and ethnicity. Because of the exploratory nature of the study, no a priori power analyses were conducted to determine sample size. Participants' age ranged between 18 and 50 years (M = 19.79, SD = 2.93) with 69% identifying as female. Most participants identified as Latino (42.7%), followed by Caucasian (27.8%), Multi-ethnic (12%), African American (10.5%), and Asian (7%). Participants completed the online survey through SurveyMonkey and received partial credit toward an experimental course requirement. This study was approved by the Institutional Review Board (IRB) at UTSA, and informed consent was obtained from all individual participants included in the study prior to study onset.

### Measures

#### Delay discounting

Delay discounting was measured using a monetary gains questionnaire (1). Participants made 27 separate choices between smaller, immediate rewards or larger, delayed rewards. For example, they chose between $25 right now and $60 in 14 days. Delays ranged from 7 to 186 days, and the larger amount ranged from $25 to 85 US dollars. Previous research shows that people discount real and hypothetical rewards at similar rates, and hypothetical rewards are a valid proxy in delay discounting research (46). For each participant, indifference points were calculated and plotted as a function of time. An indexed *k*-value indicated discounting curve steepness, which corresponded with the geometric midpoint of the ranges [see Kirby and Maraković (1)]. Minimum and maximum values were 0.00016–0.25, with higher *k*-values indicating higher delay discount rates. *k*-values were normalized using a natural logarithm function to minimize skewness. Consistency scores for each participant were calculated based on Kaplan et al. (47).

#### Social discounting

Participants completed a monetary social discounting task (8) where they made choices between a monetary amount to keep for themselves and a monetary amount to share with a person having a specific social proximity. Participants were first asked to think about a list containing 100 people, from a friend or relative at 1 to an acquaintance at 100. Social discounting was assessed at the following social proximities: 1, 2, 5, 10, 20, 50, and 100, and was always in ascending order. Up to nine choices were made for each of the seven social proximities. Choices always started with the largest amount available for the participant alone ($155) and decreased in intervals of $10 from $155 to 75 for the participant alone. The point at which a participant switched between keeping all the money for themselves and giving up money to share with the other person at a given social distance was the indifference point. For example, a participant chose to keep $135 for themself instead of allocating $75 for themselves and $75 with the other person. However, for the next choice between $125 for themselves and $75 for the other person, they chose to forgo the $125 and allocate $75 to the other person. In this case, they did not forgo $60 (135–75 = 60) but did forgo $50 (125–75 = 50). Therefore, their indifference point at that social distance was $55 [(60 + 50)/2].

Social discounting was estimated by calculating the area under the curve (AUC) for each participant using the seven indifference points with an ordinal scaling transformation [i.e., AUC_*ord*_; (48)]. Calculated AUC scores range from 0 to 1 with scores closer to 1 indicating a shallow discounting function (i.e., more sharing). Previous research has shown that people discount real and hypothetical rewards at similar rates, and that hypothetical rewards are a valid proxy for social discounting research (49).

#### Health behaviors

Participants self-reported on two yes or no questions about their health behaviors including if they have ever been tested for STIs and/or drink alcohol. If participants endorsed that they drink alcohol, they were asked to indicate how many drinks per week. Additionally, exercise frequency was also assessed *via* self-report, in both number of days per week (never, 1–2, 3–4, 5+ days) and hours per day (<30, 30–60 min, 1–3 h, 3+ h).

### Analyses

Two attention checks indicated careless responding (e.g., “I will select ‘Agree' for this answer to show that I am paying attention”). Twenty-four people were excluded from analysis for failing the attention checks [23 of these 24 also had inconsistent scores on the social discounting task (see below), validating attention check use]. Nine participants were excluded for having delay discounting consistency scores lower than 75% (47). Ninety-seven people (24.6% of the total sample) were excluded for non-systematic responding on the social discounting measure using the algorithm described by Johnson and Bickel (50). These exclusions resulted in a total of 265 participants included in analysis.

A Spearman's rho correlation between discounting measures determined any relationship between these measures (9, 12). A series of regression analyses were used to determine if delay discounting, social discounting, ethnicity, and gender predicted STI testing, alcohol use and exercise frequency. A binary logistic regression was used to determine which factors predict whether a person has been tested for STIs. To account for the large proportion of participants who did not consume alcohol, a zero-inflated Poisson regression was used to predict the number of alcoholic beverages consumed per week. Finally, an ordinal logistic regression was used to examine which of the analysis variables predicts exercise frequency among the four ordered categories (never, 1–2, 3–4, 5+ days). Robustness checks were conducted by running the analyses with all excluded respondents. The results were mostly unaffected with the exception of the analysis predicting the number of alcoholic beverages consumed per week. However, the analyses with exclusions are reported in interest of being conservative and accounting for nonsystematic responding on the social discounting measure.

## Results

### Demographic measures and discount rate correlations

One hundred and ninety-four participants (73%) indicated they were sexually active with at least one partner. A total of 97 participants (36.6% of the entire sample, 50% of all sexually active participants) indicated they had previously taken an STI test, with 16 of the 97 (16.5%) reporting testing positive for an STI. One hundred and fifty participants (56.6%) indicated they drink alcohol, with counts ranging from 1 to 25 drinks per week (M = 3.07, SD = 3.28, Median = 2). Most participants (245, 90.6%) indicated that they exercised at least once per week with 26.8% of all participants reporting exercising 1–2 days per week, 37.4% reporting 3–4 days per week, 26.4% reporting 5 or more days per week. For those individuals reporting exercise, 34 (12.8%) exercised for <30 min total on days when they exercised, 122 (46%) exercised 30–60 min, 78 (29.4%) exercised 1–3 h, and 6 (2.3%) exercised 3 or more hours. Table 1 contains additional information on participant demographics, health behavior endorsement, and discounting behaviors.

**Table 1:** Descriptive statistics.

	**Full sample**	**Latino/a**	**Caucasian**	**Multi-ethnic**	**African American**	**Asian**
*N*, %	265, 100%	114, 43%	71, 26.8%	34, 12.8%	26, 9.8%	20, 7.5%
**Gender**
Female	186, 70.2%	82, 71.9%	48, 67.6%	23, 67.6%	23, 88.5%	10, 50%
Male	79, 29.8%	32, 28.1%	23, 32.4%	11, 32.4%	3, 11.5%	10, 50%
**Alcohol consumption**
Yes	150, 56.6%	61, 53.5%	50, 70.4%	25, 73.5%	8, 30.8%	6, 30%
No	115, 43.4%	53, 46.5%	21, 29.6%	9, 26.5%	18, 69.2%	14, 70%
**Sexually active**
Yes	194, 73.2%	89, 78.1%	53, 74.6%	27, 79.4%	16, 61.5%	9, 45%
No	71, 26.8%	25, 21.9%	18, 25.4%	7, 20.6%	10, 38.5%	11, 55%
**STI testing**
Yes	97, 36.6%	37, 32.5%	27, 38%	18, 52.9%	12, 46.2%	3, 15%
No	168, 63.4%	77, 67.5%	44, 62%	16, 47.1%	14, 53.8%	17, 85%
**Exercise: Days per week**
Never	25, 9.4%	10, 8.8%	5, 7%	3, 8.8%	5, 19.2%	2, 10%
1–2	71, 26.8%	31, 27.2%	17, 23.9%	10, 29.4%	7, 26.9%	6, 30%
3–4	99, 37.4%	45, 39.5%	29, 40.8%	11, 32.4%	9, 34.6%	5, 25%
5–7	70, 26.4%	28, 24.6%	20, 28.2%	10, 29.4%	5, 19.2%	7, 35%
**Exercise: Time per day**
No exercise	25, 9.5%	10, 8.8%	5, 7%	3, 8.8%	5, 19.2%	2, 10%
<30 min	34, 12.8%	15, 13.2%	9, 12.7%	4, 11.8%	4, 15.4%	2, 10%
30–60 min	122, 46%	51, 44.7%	35, 49.3%	17, 50%	10, 38.5%	9, 45%
1–3 h	78, 29.4%	34, 29.8%	20, 28.2%	10, 29.4%	7, 26.9%	7, 35%
3+ h	6, 2.3%	4, 3.5%	2, 2.8%	0	0	0
**M, SD, mode**
Age	19.82, 3.28, 19	19.49, 2.03, 18	20.69, 5.31, 19	19.79, 2.46, 18	19.12, 1.28, 19	19.6, 1.93, 19
Alcoholic drinks per week	3.07, 3.28, 2	1.75, 3.35, 0	2.11, 2.66, 0	2.29, 3, 0	0.5, 0.91, 0	0.7, 1.26, 0
Delay discounting	0.04, 0.05	0.04, 0.05	0.27, 0.04	0.37, 0.05	0.51, 0.07	0.05, 0.07
Social discounting	0.49, 0.27	0.5, 0.27	0.48, 0.27	0.53, 0.25	0.46, 0.52	0.49, 0.31

Delay discounting consistency scores ranged from 0.78 to 1.00 (M = 0.95, SD = 0.05). A within subjects ANOVA with a Greenhouse-Geisser correction between the three magnitudes (small, medium, and large) for delay discounting showed a magnitude effect [*F*_(1.90, 729.77)_ = 162.72, *p* < 0.001], supporting measure validity within this sample. The natural log *k*-values, or delay discount rates, (M = −4.32, SD = 1.61) were significantly negatively correlated with social discount rates (M = 0.49, SD = 0.27, *r* = −0.162, *p* = 0.008) measured *via* AUC_*ord*_, using a Spearman's rho correlation to account for the skewed social discounting distribution. That is, as delay discounting increased, sharing decreased (i.e., selfishness increased). When comparing across ethnicities in the sample, Latino Americans (*r* = −0.100, *p* = 0.291, *n* = 114), African Americans (*r* = −0.454, *p* = 0.020, *n* = 26), Caucasians (*r* = −0.067, *p* = 0.580, *n* = 71), and Multi-ethnic participants (*r* = −0.338, *p* = 0.051, *n* = 24) demonstrated negative weak to moderate relationships between delay and social discount rates. Associations between discount rates were only significant among African Americans. Asian Americans demonstrated a positive non-significant relationship between discount rates (*r* = 0.383, *p* = 0.096, *n* = 20). Between subject ANOVAs were conducted to examine differences among ethnic groups in delay discounting and social discounting respectively. A significant difference among ethnic groups was found for delay discounting [*F*_(4, 260)_ = 2.51, *p* = 0.04], but not social discounting. A Tukey *post-hoc* test found no significant pairwise comparisons for delay discounting among ethnic groups. Table 2 provides means and standard deviations for both delay and social discounting by ethnic group. Due to weak to moderate correlations among the analysis variables, variance inflation factors (VIF) were examined as a diagnostic of multicollinearity. All analysis models demonstrated low VIF values (<2) indicating multicollinearity was not present (51).

**Table 2:** Discounting means and standard deviations by ethnic group.

		**Delay discounting**		**Social discounting**	
**Ethnicity**	***n***	***M***	***SD***	***M***	***SD***
Latino/a	114	−4.42	1.66	0.5	0.27
Caucasian	71	−4.68	1.76	0.48	0.27
Multi-ethnic	34	−3.93	1.25	0.53	0.25
African American	26	−3.79	1.56	0.46	0.27
Asian	20	−3.89	1.56	0.49	0.31

### Discount rates and STI testing

**Figure 1:**
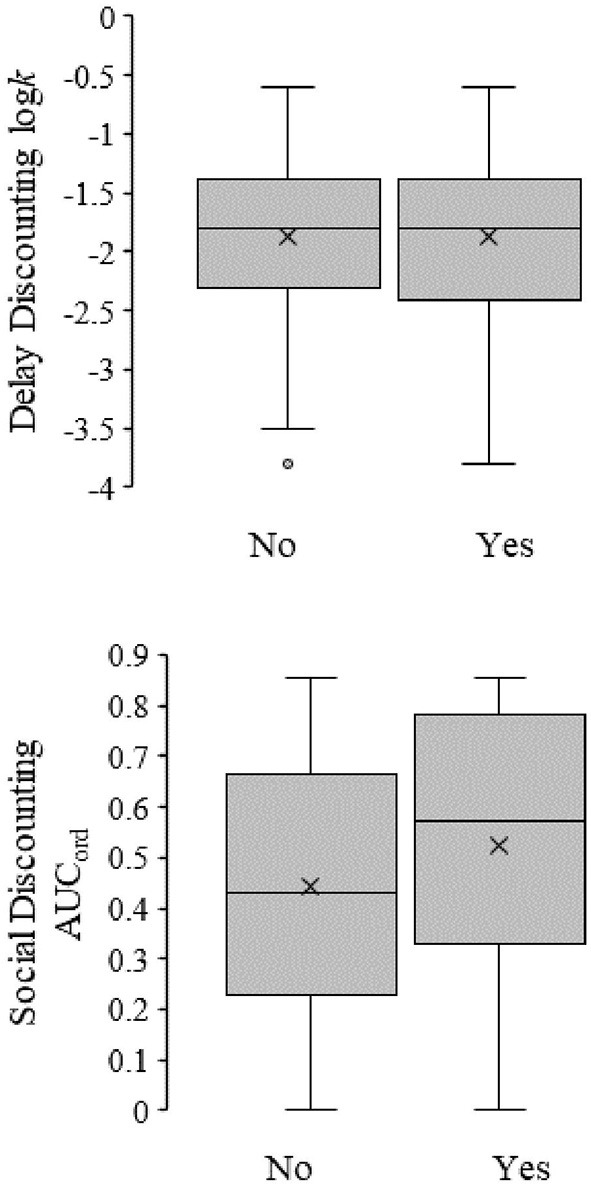
Box-and-whisker plots for the relationship between delay (top figure) and social discount (bottom figure) rates and STI testing. The lower and upper bound for each box represents the first and third quartiles for discount rates, respectively. The horizontal lines bisecting each box represents the median discount rates. The X within each box represents the mean discount rate. Vertical lines extending above and below the box represent 1.5 times each interquartile range. Circles represent outliers. The left box represents individuals self-reporting no STI test (*n* = 168), whereas the right box represents individuals self-reporting being tested for STIs (*n* = 97).

A binary logistic regression was used to determine if delay discounting, social discounting, ethnicity, and gender predicted if participants had been tested for STIs [$\chi_{\left(7\right)}^{2}$ = 16.395, *p* = 0.021]. Social discount rates and Multi-ethnicity significantly added to the model, but delay discount rates (*p* = 0.721) did not (see Figure 1—top graph). Figure 1 (bottom graph) shows that participants with higher rates of social discounting (i.e., greater sharing or less selfishness) were more likely to have been tested for STIs (B = 1.211, SE = 0.494, OR = 3.359, *p* = 0.014). Ethnicity also contributed significantly to the model. When compared to Hispanic Americans, those who were Multi-ethnic were less likely to be tested for STIs (B = −0.919, SE = 0.405, OR = 0.398, *p* = 0.023).

### Discount rates and alcohol use

**Figure 2:**
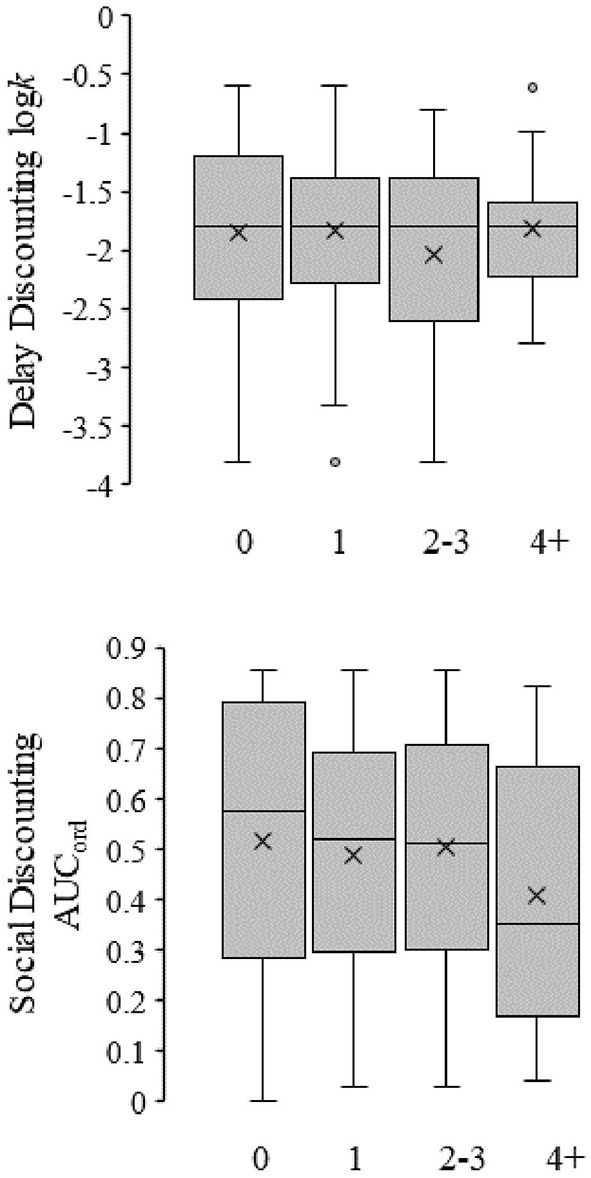
Box-and-whisker plots for the relationship between delay (top figure) and social discount (bottom figure) rates and alcohol use. Symbols are the same as in Figure 1. The leftmost box represents individuals self-reporting no alcohol use (*n* = 115), whereas subsequent boxes represent individuals self-reporting drinks per week in ascending order (*n*'s; 1 = 54, 2–3 = 53, and 4+ = 39).

A zero-inflated Poisson regression with delay and social discount rates, gender, and ethnicity was used to predict the number of alcoholic beverages consumed per week [$\chi_{\left(16\right)}^{2}$ = 30.384, *p* < 0.001]. This analysis technique was used due to the large proportion of participants who did not consume alcohol. Ethnicity and gender significantly predicted alcohol consumption. As shown in Figure 2 neither delay (top graph; *p* = 0.761) nor social discounting (bottom graph; *p* = 0.172) significantly predicted alcohol consumption. When compared to Hispanic Americans, African Americans (B = −1.11, SE = 0452, IRR = 3.116, *p* = 0.014) consumed less alcohol per week. Additionally, females (B = −0.486, SE = 0.105, IRR = 0.589, *p* < 0.001) consumed less alcohol per week than males.

### Discount rates and exercise

**Figure 3:**
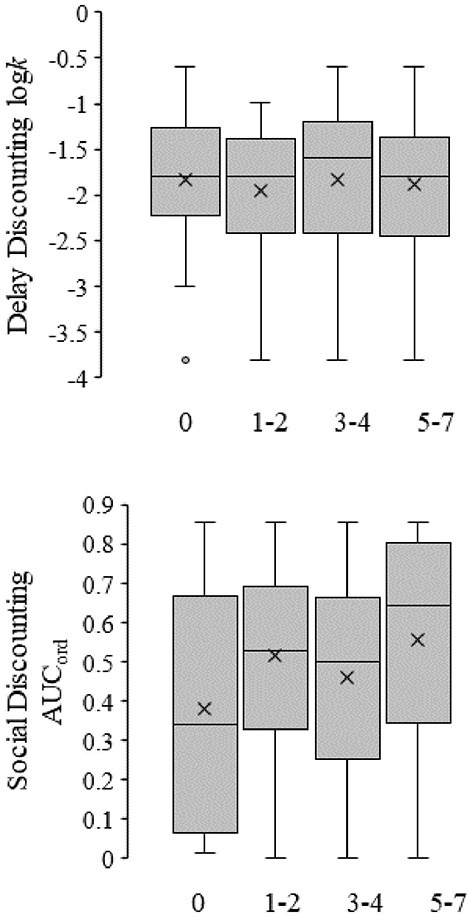
Box-and-whisker plots for the relationship between delay (top figure) and social discount (bottom figure) rates and amount of exercise per week. Symbols are the same as in Figures 1, 2. The leftmost box represents individuals self-reporting no exercise (*n* = 25), whereas subsequent boxes represent individuals self-reporting increasing amounts of exercise per week in ascending order (*n*'s; 1–2 = 71, 3–4 = 90, and 5–7 = 70).

An ordinal logistic regression modeled whether delay and social discount rates, gender, and ethnicity predicted days per week spent exercising. Days spent exercising fell into four ordered response categories: never, 1–2, 3–4 days, and 5 or more days. The predictors significantly improved model fit over the intercept only model [$\chi_{\left(7\right)}^{2}$ = 15.514, *p* = 0.029]. As shown in Figure 3 neither delay (top graph; *p* = 0.655) nor social discount rates (bottom graph; *p* = 0.079) were significant predictors of days per week spent exercising. Gender significantly predicted days spent exercising indicating that females exercised less frequently than males (B = −0.740, SE = 0.252, OR = 0.476, *p* = 0.003).

In addition to modeling days per week spent exercising, an ordinal logistic regression modeled whether delay and social discount rates, gender, and ethnicity predicted time spent exercising. Time spent exercising fell into four ordered response categories: <30, 30–60 min, 1–3, and 3 h or more. The predictors significantly improved model fit over the intercept only model [$\chi_{\left(7\right)}^{2}$ = 14.732, *p* = 0.039]. While neither delay discount rate (*p* = 0.510) nor social discount rate (*p* = 0.893) were significant predictors, gender significantly predicted time spent exercising, with females demonstrating less time exercising than males (B = −1.021, SE = 0.275, OR = 0.360, *p* < 0.001).

## Discussion

Previous research has demonstrated that choice tasks such as delay and social discounting are meaningfully associated with various health concerns such as alcohol use, sexual behaviors, and body weight (52–56). That is, those individuals demonstrating higher delay discount rates may also demonstrate more deleterious health behaviors (2). The current results showed that social discounting, but not delay discounting, was predictive of one of three health behaviors that varied on sociality in an ethnically diverse sample. Specifically, social discount rates only predicted STI testing rates by ethnicity. In this case, increased selfishness was associated with a decreased likelihood to be tested for STIs (see Figure 1 bottom graph).

### Correlations between discount rates

Previous researchers have reported significant correlations between delay and social discount rates (9, 12). In the current sample, delay and social discounting were significantly negatively correlated (*r* = −0.162), replicating previous research. The current correlation coefficient was more like (12) (*r* = −0.14), relative to (9) (*r* = 0.25, *r* = 0.28; using *k-* and *s-*values rather than *k*-values and AUC_ord_ like the current paper, accounting for the negative relationship). Methodologically, Jones and Rachlin's (9) used larger delay and monetary values (up to 5 years and $1,000) relative to both this study and Wainwright et al. The current study also showed different correlation coefficients for different ethnicities. Unfortunately, ethnicity data was not reported by Jones and Rachlin (9). Future researchers should consider reporting discount rate correlations based on ethnicity. Because delay discounting can be conceptualized as a special case of social discounting, where an individual shares with themselves as a function of time (57), social discounting differences by ethnicity may give us a way to measure how structural and/or environmental differences impact selfishness, as well as impulsivity.

### Discounting and STI testing

There was a significant relationship between social discount rates and STI testing. In this case, individuals who were more selfish were less likely to have been tested for STIs, consistent with our hypothesis. These results suggest that individuals may be more likely to be tested for STIs due to concern for the health of others. Previous research supports this notion in that positive social norms toward STI testing significantly predicted intentions to be tested for STIs in the next month among university students (58). Thus, the overall research trend illustrates how one's social context may influence one's intentions and behaviors regarding testing for STIs. Given the consistent relationship between social norms, social discounting and STI testing, it may be worthwhile to determine if social norms and social discounting are correlated, regarding STI testing. Determining if social discounting adds to predicting intentions to be tested for STIs is important information for any STI testing intervention aiming to increase STI testing.

Figure 1 (top graph) shows that there was no significant relationship between delay discount rate and STI testing. Previous research has demonstrated associations between delay discount rate and sexual risk-taking (41), but these results had not been extended to STI testing until now. STI testing is a behavior that can occur well before the sexual act itself. This delay between testing and the sexual act would imply less delay discounting for those individuals engaging in STI testing. However, the current results suggest increased selfishness is better associated with decreased STI testing, relative to lower delay discount rates. That is, delay discounting perhaps plays a larger role when the sexual act is more proximal, whereas selfishness plays a larger role when the sexual act is more distal.

The current results showed that ethnicity was also a significant predictor for STI testing. Previous investigations in to discounting and sexual behavior [e.g., (41)] included relatively homogeneous samples and treated sexual behaviors on an ordinal scale while this investigation used a binary scale for STI testing. However, a binary measure for this health outcome may not be sensitive enough. In addition, there may be individuals who are risk averse that do not engage in risky sexual behavior, and therefore do not engage in STI testing. The current binary STI testing measure was exploratory in regard to a potential relationship with two discounting measures, based on previous research (18–20). That such a simple measure was significantly related to social discounting suggests a more comprehensive measure for risky sexual behavior should also yield significant associations with discount rates. Therefore, future studies should measure whether STI testing occurs, sexual behavior as frequency measures, and when STI testing typically occurs (pre- and/or post-intercourse) to determine if one or both are associated with different discount rates.

### Discounting and alcohol use

We hypothesized that both increased delay discount rates and social discount rates (i.e., increased selfishness) would predict increased alcohol use. However, the results did not support our hypothesis as neither delay nor social discounting predicted alcohol consumption. However, ethnicity was a significant predictor for alcohol use. Similar research has also failed to find relationships between discounting behaviors and alcohol consumption, underscoring the need to include more diverse samples in health behavior research (29).

Failure to detect relationships between alcohol consumption and discounting behaviors may be associated with sample diversity. Consistent with the current findings, previous research with similar population demographics failed to find a relationship between alcohol use and social discounting tasks when alcohol use was dichotomized as either drinking or not (29). In addition, there was no significant association between delay discounting and other health behaviors, such as TWD, even though alcohol use significantly increased with TWD (28). Perhaps the typical relationship between alcohol consumption changes as the experimental sample becomes increasingly diverse. That is, if ethnicity consistently predicts relationships between alcohol consumption and discount rates for select ethnicities (e.g., Caucasian), then any population sample with an overrepresentation of Caucasian individuals will show that discount rates significantly predict alcohol consumptions, whereas those with an underrepresentation of Caucasian individuals will not. Unfortunately, ethnicity was not reported in Gowin et al. (25), which included 793 individuals. In Petry (26) ~80% of participants were Caucasian from a total sample of 46, making any ethnic comparison extremely difficult. Most other subsequent studies have had similarly small sample sizes [see (4) for meta-analysis]. Whether the null finding for delay discounting and alcohol consumption is a function of demographic differences should be explored in future research.

### Discounting and exercise

There were no statistically significant relationships between exercise and delay discount rates, nor exercise and social discount rates (see Figure 3). The current results are inconsistent with the experimental results for Sofis et al. (35) who found that increased delay discount rates reduced physical activity rates. However, there was a significant association for gender with exercise in the current sample. Sofis et al. (35) only included females and recruited based on self-reported weight problems. The sample in the current investigation was larger, contained males, and included many individuals who exercised very often (see Table 1). This is similar to Rasmussen et al. (34) who did not find a significant association between exercise and delay discounting.

To the researchers' knowledge, this was the first investigation assessing the relationship between social discount rates and exercise. Given the relatively high rate of exercise behavior reported, it is likely that the results obtained from the sample in this investigation are not generalizable to populations that do not normally exercise (i.e., non-college populations). A study investigating social discounting and health outcomes with an MTurk sample found that social discount rates were associated with obesity (59), which is partially a consequence of low (or no) exercise. Thus, the relationship between social discount rates and exercise may vary depending on how regularly a certain population engages in physical activity.

### Ethnic differences

Discounting and health behavior research has been conducted with predominately homogeneous samples (21, 26, 32–34, 40, 41). The current investigation, as well as a few previous studies, have found evidence that different ethnicities exhibit different patterns of discounting behavior (42, 43). In addition, both ethnicity and social discounting significantly predicted STI testing in the current study. More importantly, ethnicity was always a significant predictor for each of the three health behaviors measured, despite the association between the health and discounting behaviors only being statistically significant for one of those health behaviors. It is worth noting that the *post-hoc* analysis found no significant results when comparing across ethnicities. The current *post-hoc* analysis included ten comparisons, making alpha more conservative per comparison. Perhaps no significant differences were found due to a lack of power. More research on discounting behaviors in ethnically diverse samples are needed to properly ascertain potential ethnic differences. The ubiquity of health differences between ethnicities in the US (39) suggest that including a range of different ethnicities will be crucial for accurately describing differential associations between health behaviors and discount rates.

### Limitations and future directions

Some limitations should also be considered. First, all health behaviors in this investigation were indicated *via* self-report and are susceptible to memory limitations and/or response biases. Second, while the sample in this investigation was ethnically diverse, the current sample contains relatively small percentages of Multi-ethnic, African American, and Asian participants. Greater proportions of ethnic minorities or focusing analysis to a single ethnic minority population in future research would help to further highlight differences and similarities in discounting and health behaviors across ethnicities. Third, the findings in this research are necessarily limited due to its cross-sectional design and categorical analyses. A cross-sectional design makes it difficult to ascertain the causal nature for the relationship between discounting and health behaviors. Future research should address these limitations in this investigation by using more objective health indicators such as blood alcohol content, heart rate data from fitness tracking devices, patient reports for STI testing, as well as more sophisticated modeling techniques, such as structural equation modeling, to strengthen arguments for causality and resolve contradictory results. Fourth, risk for each of the three health behaviors, or concomitant behaviors (e.g., STI risk, obesity risk), was not assessed. Results from Romanowich et al. (28) showed that when participants rate a behavior as less risky (i.e., <50 out of 100), there is less of a chance of finding a discounting—health behavior association. Lastly, because of the exploratory nature of the current study, no a priori power analysis was conducted to decide participant sample size. The results of the current study, especially with regards to STI testing, should help facilitate adequately powered future studies to test associations with discounting rates.

The current study showed a novel relationship between social discount rates and STI testing for an ethnically diverse college sample. Ethnicity was a significant predictor in all three health behavior models. These findings demonstrate the importance of using an ethnically diverse sample when conducting research on discounting and health behaviors. Public health programs could profitably use this information when designing interventions and educating diverse college student populations on how STI testing affects themselves and those around them.

## Data availability statement

The raw data supporting the conclusions of this article will be made available by the authors, without undue reservation.

## Ethics statement

The studies involving human participants were reviewed and approved by University of Texas at San Antonio Institutional Review Board. Written informed consent for participation was not required for this study in accordance with the national legislation and the institutional requirements.

## Author contributions

NB conceived and planned the data analyses with the help of KW. KW and PR carried out the study. NB, KW, RT, and VL contributed to analyzing the raw data and the method and discussion section. NB, KW, and PR contributed to interpretation of results. NB took the lead on manuscript writing and contributing to every section. NB, KW, and RT contributed write up of the results and supervised with edits throughout. PR contributed to portions of the introduction and discussion sections. NB, KW, RT, VL, and PR provided edits and added content throughout the manuscript. All authors contributed to the article and approved the submitted version.

## Conflict of interest

The authors declare that the research was conducted in the absence of any commercial or financial relationships that could be construed as a potential conflict of interest.

## Publisher's note

All claims expressed in this article are solely those of the authors and do not necessarily represent those of their affiliated organizations, or those of the publisher, the editors and the reviewers. Any product that may be evaluated in this article, or claim that may be made by its manufacturer, is not guaranteed or endorsed by the publisher.
